# A Rapid, Semi-Quantitative Assay to Screen for Modulators of Alpha-Synuclein Oligomerization *Ex vivo*

**DOI:** 10.3389/fnins.2015.00511

**Published:** 2016-01-19

**Authors:** Marion Delenclos, Teodora Trendafilova, Daryl R. Jones, Simon Moussaud, Ann-Marie Baine, Mei Yue, Warren D. Hirst, Pamela J. McLean

**Affiliations:** ^1^Department of Neuroscience, Mayo ClinicJacksonville, FL, USA; ^2^Pfizer Neuroscience Research UnitCambridge, MA, USA; ^3^Mayo Graduate School, Mayo ClinicJacksonville, FL, USA

**Keywords:** alpha-synuclein, oligomers, protein-fragment complementation, bioluminescence, viral vector model

## Abstract

Alpha synuclein (αsyn) aggregates are associated with the pathogenesis of Parkinson's disease and others related disorders. Although modulation of αsyn aggregation is an attractive therapeutic target, new powerful methodologies are desperately needed to facilitate *in vivo* screening of novel therapeutics. Here, we describe an *in vivo* rodent model with the unique ability to rapidly track αsyn-αsyn interactions and thus oligomerization using a bioluminescent protein complementation strategy that monitors spatial and temporal αsyn oligomerization *ex vivo*. We find that αsyn forms oligomers *in vivo* as early as 1 week after stereotactic AAV injection into rat substantia nigra. Strikingly, although abundant αsyn expression is also detected in *striatum* at 1 week, no αsyn oligomers are detected at this time point. By 4 weeks, oligomerization of αsyn is detected in both striatum and substantia nigra homogenates. Moreover, in a proof-of-principle experiment, the effect of a previously described Hsp90 inhibitor known to prevent αsyn oligomer formation, demonstrates the utility of this rapid and sensitive animal model to monitor αsyn oligomerization status in the rat brain.

## Introduction

Under pathological conditions alpha-synuclein (αsyn) can misfold and aggregate into insoluble deposits that accumulate in cells to toxic levels. The conversion of αsyn from its functional conformation into a misfolded and toxic conformation constitutes the basis of a group of diseases known as synucleinopathies which include Parkinson's disease (PD), multiple system atrophy, and dementia with Lewy bodies (Goedert and Spillantini, 1998; Kim et al., 2014). Several lines of evidence demonstrate a strong association between αsyn aggregation and neurodegeneration (Irizarry et al., 1998; Chartier-Harlin et al., 2004; Winner et al., 2011). Fibrillar forms of αsyn are the major component of glial cytoplasmic inclusions, Lewy bodies (LBs), and Lewy neurites, defined as intracytoplasmatic inclusions and considered the pathological hallmarks of synucleopathies (Forno, 1996; Spillantini et al., 1997; Halliday et al., 2011). Normally, αsyn is a soluble, presynaptic protein that may exist as a natively unfolded monomer or a functional tetramer (Bartels et al., 2011; Wang et al., 2011; Fauvet et al., 2012; Burré et al., 2013; Gurry et al., 2013; Selkoe et al., 2014; Dettmer et al., 2015a,b). The processes that lead to pathological aggregate formation occur through the formation of several soluble oligomeric intermediates that mature into the insoluble amyloid fibrils found in LBs. It has been proposed that prefibrillar forms of αsyn are the disease-causative toxic species, while the insoluble fibrils might represent a protective pathway for surviving cells (Ross and Poirier, 2004; Paleologou et al., 2009; Winner et al., 2011; Kalia et al., 2013).

Although the mechanisms of αsyn-induced toxicity remain unclear, inhibition of oligomerization represents an attractive therapeutic approach. Such a strategy requires the availability of powerful cellular and rodent models. Current methods for detection of αsyn oligomers are challenging due to their dynamic nature and sensitivity to external conditions (Uversky, 2003; Drescher et al., 2012; Gurry et al., 2013) Thus, far, oligomer studies have used indirect methods and biochemical techniques that prohibit the study of αsyn oligomerization in a live cellular environment or in intact brain. More recently, protein fragment complementation assay (PCA) strategies that allow the detection of αsyn-αsyn interactions using fluorescence or bioluminescent reporters, have been widely developed and have been successfully applied to monitor αsyn oligomers not only in living cells (Outeiro et al., 2008; Putcha et al., 2010; Danzer et al., 2012) but also in rodent brain (Dimant et al., 2013, 2014; Aelvoet et al., 2014; McFarland et al., 2014).

Here, we generated and characterized a viral vector rodent model where humanized *Gaussia princeps* luciferase (hGluc) is used as a surrogate reporter of αsyn oligomerization *in vivo* in a fast, sensitive, and semi quantitative assay. We demonstrate that this rodent model can be utilized to track αsyn oligomerization *in vivo* and validate the potential use of the model by testing a novel Hsp90 inhibitor compound, known to reduce αsyn oligomerization *in vitro*, on our *in vivo* bioluminescence read out.

## Materials and methods

### Viral vector production

The viral vectors pAAV-CBA-synuclein-LUC1-WPRE (SL1) and pAAV-CBA-SYNUCLEIN-luc2-WPRE (SL2) were constructed by inserting the human SNCA cDNA (h αsyn) fused to either the N-terminus half of humanized *G. princeps* luciferase (hGluc) (Tannous et al., 2005) or the C-terminus half of hGluc, into the EcoRV and NheI sites of the pAAV-CBA-WPRE. Viral vector pAAV-CBA-*G. princeps* luciferase was constructed by inserting the full length of hGluc gene into the XhoI and NheI sites of pAAV-CBA-WPRE vector. Adeno-associated virus (AAV) serotype 2/8 was produced by plasmid transfection with helper plasmids in HEK293T cells. Forty-eight hours later, the cells were harvested and lysed in the presence of 0.5% sodium deoxycholate and 50μ/ml Benzonase (Sigma-Aldrich, St. Louis, MO) by freeze-thawing, and the virus was isolated using a discontinuous iodixanol gradient. The genomic titer of each virus was determined by quantitative PCR.

### Surgical procedure

Adult Female Sprague Dawley rats (225–250 g, Harlan, USA) were housed and treated in accordance with the NIH Guide for Care and Use of Laboratory animals. All procedures were approved and conducted in accordance with the Mayo Clinic Institutional Animal Care and Use committee. Rats were housed 3 per cage with ad-libitum access to food and water during a 12 h light/dark cycle. Surgery was conducted under 2% isoflurane anesthesia mixed with O2 and N2 using a stereotaxic frame and a 10 μl Hamilton syringe fitted with a 30 gauge needle. The scalp was exposed and a unilateral injection targeting the substantia nigra (SN) was performed at coordinates 5.2 mm posterior and 2 mm lateral to bregma, and 7.2 mm ventral relative to dura. AAV8 vectors were normalized by titer and volume, resulting in injection of an equal amount of genomes per copy per vector. Two microliter of a mixture of two viruses (SL1 8.10^12^ g/ml + SL2 8.10^12^ g/ml) were injected at a rate of 0.4 μl/min using a microinjection pump (Stoelting Co, Wood Dale, IL). Control animals were injected with one virus only (2 μl of AAV8-SL2 at 16.10^12^ genome/ml), or received one injection of 1 μl of SL1 (8.10^12^ g/ml) in the SN of the left hemisphere (ML: −2 mm) and one injection of 1 μl of SL2 (8.10^12^ g/ml) in the SN of the other hemisphere (ML: 2 mm). At the end of injection the needle remained in place for 5 min before being slowly retracted. Animals were then sutured with metal clips and monitored until fully recovered.

### Tissue processing

For histological analyses, animals were deeply anaesthetized at 1 and 4 weeks post-injection with pentobarbital and perfused transcardially with ice-cold 0.9% saline, followed by 4% paraformaldehyde (PFA). Brains were removed and post-fixed for 4 h in the same solution and were then transferred overnight to 25% sucrose solution for cryoprotection. The brains were cross-sectioned using a freezing stage sliding microtome (Leica SM2010R, Germany) at 40 μm in the coronal plane. Brains from a subset of animals from 1 to 4 weeks post injection were harvested fresh without fixation. The two hemispheres were separated and the striatum (STR) and midbrain from both sides were quickly dissected on ice and used directly for biochemical analysis.

### Immunohistochemistry

Immunohistochemical analysis was performed on free-floating sections (40 μm) using primary antibodies against tyrosine hydroxylase (TH) (1:3000, MAB318, Millipore), and hαsyn (1:2000, 4B12, specific antibody to human form of αsyn, Covance). Sections were washed with phosphate buffer saline (PBS) before each incubation. After the initial wash, the sections were quenched for 20 min at RT with PBS solution, supplemented with 3% H_2_O_2_ (v/v) (Sigma-Aldrich), and 10% Methanol (v/v) (Pharmaco-Aaper) and were then blocked with 5% goat serum (Life technologies, Carlsbad, CA, USA) in T-PBS (0.25% Triton dissolved in PBS). The sections were incubated overnight at RT with one of the indicated antibodies, diluted in 2.5% goat serum and T-PBS. After washing, the brain slices were treated with 1:200 dilution of the appropriate biotinylated secondary antibody (Vector Laboratories, Burlingame, CA, USA) in 1% goat serum and T-PBS. Thereafter, the sections were rinsed and incubated with avidin-biotin-peroxidase complex (ABC peroxidase kit, Thermo Scientific). The staining was visualized by 3,3-diaminobenzidine (DAB; Sigma-Aldrich) used as a chromagen and activated by 1% H_2_O_2_. The sections were then mounted on gelatin-coated slides, dehydrated with alcohol, cleared with xylene and coverslipped with DePex mounting medium (Sigma-Aldrich).

### Stereology

Nigrostriatal cell loss was assessed using an unbiased stereology method using the optical fractionator principle as described previously (West et al., 1991). Lower magnification was used to trace the region of interest (SN in each hemisphere) at all levels in the rostro-caudal axis in 9–10 sections per animals. TH+ cells in the ventral tegmental area (VTA) were excluded. The counting was performed using the Stereo Investigator software (MBF bioscience). In addition, the counting frame size was adjusted so that ~100–200 TH+ positive cells were counted in each side of the SN. The estimation of total neuron numbers was performed by using the optical fractionator formula and coefficient of error < 0.1 was accepted (West, 1999).

### Densitometry

Images of forebrain were captured using the Aperio slide scanner (Aperio, Vista, CA, USA). The optical density of striatal TH+ fibers was measured using ImageJ software (Version 1.46, National Institutes of Health) at four coronal levels according to the rat brain atlas of Paxinos and Watson (6th edition, 2007): AP: +1.2,+ 0.8, 0.0 and −0.4 mm relative to bregma. The measured values were corrected for non-specific background staining by subtracting values obtained from adjacent cortical areas. The data are expressed as a percentage of the corresponding area from the intact side.

### *Ex vivo* luciferase complementation assay

Fresh STR and SN were homogenized briefly in 200 μl cold PBS buffer using a Teflon pestle. One-hundred microliter of the homogenate was transferred to a 96 well plate (Costar, Corning, NY, USA) and the remainder was preserved for biochemical analysis. Luciferase activity from αsyn-αsyn interaction was measured in an automated plate reader (Victor 3 multilabel counter, Perkin Elmer, Waltham, MA, USA) at 480 nm following the injection of 100 μl of the substrate coelenterazine (40 μm, NanoLight tech, AZ, USA) with a signal integration of 2 s.

### Human αsyn elisa

Quantitative analysis of hαsyn concentration in rat tissue was performed using hαsyn specific ELISA (#KHBOO61, Life tech) according to the manufacturer's instructions. Absorbance at 450 nm is directly proportional to the concentration of hαsyn present in the original specimen. αSyn concentrations were normalized to the total amount of protein in the homogenate. Protein content was measured using Bicinchoninic acid assay (BCA) (Thermo Fisher Scientific, Waltham, MA, USA).

### Western blotting

Striatal and midbrain samples were suspended in RIPA buffer (Millipore, 20-188) containing protease inhibitor (Roche Diagnostics), homogenized on ice, and centrifuged at 13,000 × g for 10 min. Protein concentration of each lysate was determined by BCA. Twenty microgram proteins was loaded on a denaturing 4–12% Bis- Tris gradient gel (Nupage, Novex Gel, Life tech) and run according to the manufacturer's instructions. Subsequently, separated protein was transferred to polyvinylidene difluoride (PVDF) membrane, blocked in 5% non-fat milk in TBS-T, and immunoblotted for Hsp70 (1:5000, rabbit, ADI-SPA-812, Enzo). Anti-actin (1:10000, rabbit polyclonal, A2668, Sigma-Aldrich) was used as a loading control. All membranes were then incubated with a secondary antibody conjugated to HRP for 1 h at room temperature. Immunoreactivity was visualized using an ECL chemiluminescent detection Kit (Thermo Fisher Sci) and images were acquired with a CCD imaging system (LAS-4000, Fujifilm, Japan).

### SNX-9114 treatment *in vivo*

SNX-9114 (Pfizer, PF-04944733) was described previously (McFarland et al., 2014) and was dissolved in a 0.5% methylcellulose solution before use. SNX-9114 was administered at a dose of 2 mg/kg by oral gavage twice for 1 week in a volume of 1 mL. As a control, the same volume of vehicle (0.5% methylcellulose) was simultaneously administered in a separate group of rats (vehicle group). SNX-9114 administration started 2 days after the stereotactic viral injection.

### Cell cultures

H4 neuroglioma cells (ATCC, HTB-148, Manassas, USA) were maintained in Opti-MEM supplemented with 10% Fetal Bovine Serum (FBS). H4 cells were transfected with full length hGluc using Superfect transfection reagents (Qiagen, Chatsworth, CA, USA) with an equimolar ratio of plasmids according to the manufacturer's instructions.

A tetracycline-driven stable H4 cell line co-expressing hαsyn tagged with either the amino-terminal (SL1) or the carboxy-terminal fragments (SL2) of hGluc luciferase was generated and described previously (Moussaud et al., 2015). SL1SL2 cells were cultured in Opti-MEM supplemented with 10% FBS, 200 μg/ml geneticin, 200 μg/ml hygromycin B, and 1 μg/ml tetracycline to block the expression of the transgenes (SL1 and SL2). Cells were maintained at 37°C in a 95% air/5% CO_2_ humidified incubator and all the cell cultures reagents were from Life technologies.

SL1SL2 stable cells were seeded into 6-well plates at the density of 1 × 10^5^ cells/well and in a 96-well plate at the density of 3 × 10^4^ cells/well in the absence of tetracycline. Twenty-four hours later, media was replaced with FBS-free media supplemented with SNX-9114 (25, 50, 100, or 200 nM) or with DMSO alone as control. After 24 h treatment, cells in the six well plates were lysed (150 mM NaCl, 5 mM Trisbase, 0.1% TritonX, cOmplete Mini, pH 7.4) and analyzed by western blotting using antibodies against: hαsyn (1:1000,mouse, 610787, Becton Dickinson, NJ, USA), Hsp70 (1:5000, rabbit, ADI-SPA-812, Enzo life), and actin (1:10000; rabbit polyclonal, A2668, Sigma-Aldrich), whereas cells in 96 well plates were used for luciferase activity measurement as described previously (Putcha et al., 2010).

### Statistics

Data were analyzed using GraphPad Prism 6 (San Diego, CA) and are presented as mean ± standard error of the mean (S.E.M.). Statistical significance was determined using a Student's *t*-test or one-way analysis of variance with Tukey's multiple comparison *post-hoc*. *P* < 0.05 was considered significant.

## Results

### *Ex vivo* monitoring of αsyn oligomers using bioluminescent PCA

The bioluminescent αsyn PCA strategy has been widely used by our lab and others (Outeiro et al., 2008; Putcha et al., 2010; Danzer et al., 2011; Moussaud et al., 2015) to investigate αsyn oligomerization in living cells. Briefly, two hαsyn proteins fused to N- or C-terminal halves of a reporter protein can reconstitute the enzymatic activity of the reporter when αsyn-αsyn interactions occur, thus providing a readout of αsyn-αsyn interactions and oligomerization. In this study, two AAV8 vectors expressing hαsyn fused with either the N-terminus or C-terminus of hGluc (referred hereafter as AAV-SL1 and AAV-SL2) were stereotactically co-injected unilaterally into the SN of adult rats to establish the *in vivo* bioluminescent PCA. Previous studies from our lab have characterized the oligomeric species formed in the bioluminescent complementation assay as a heterogeneous population of soluble dimers and higher order multimers (Danzer et al., 2011; Dimant et al., 2013). The term oligomer is used henceforth to represent the heterogenous population.

**Figure 1:**
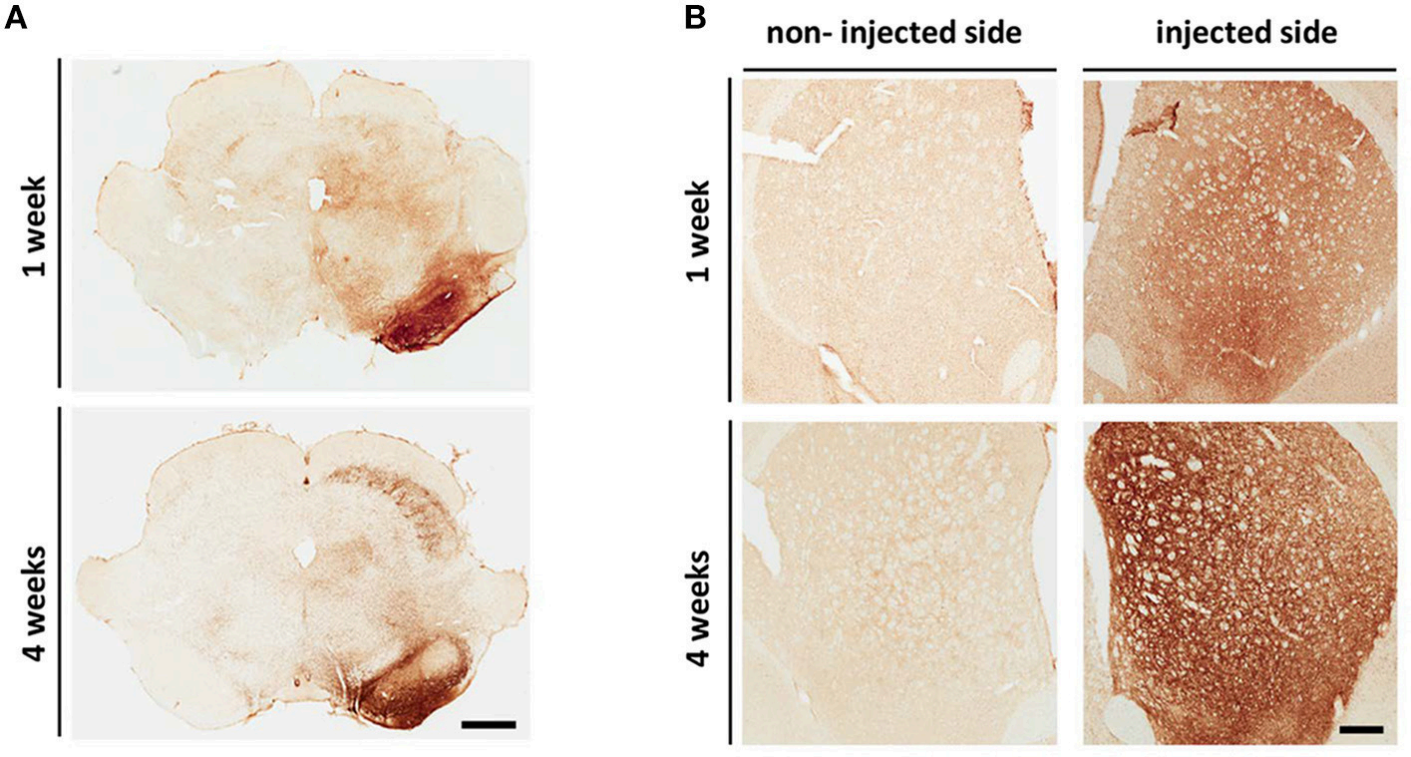
**AAV8 SL1SL2 transduction results in human αsyn over-expression along the nigrostriatal pathway. (A,B)** Representative brightfield photomicrographs of SN **(A)** and STR **(B)** at 1 and 4 weeks. Coronal sections immunostained with an antibody specific for hαsyn (clone 4B12) had expression of the transgenes in the injected SN **(A)**. The expression was also detected at the striatal level **(B)** with a more intense staining at 4 weeks, scale bar A, 1 mm; B, 500 μm.

Delivery of AAV-SL1 and AAV-SL2 into the SN of the rat produces widespread overexpression of hαsyn along the nigrostriatal pathway as revealed by histological analysis (Figures 1A,B). Immunostaining with an antibody specific for hαsyn shows numerous cell bodies in the SN expressing the transgenes at 1 week and at 4 weeks while the non-injected side is devoid of hαsyn and is used as an internal control (Figure 1A). Transport of virally transduced αsyn is indicated by the extensive network of αsyn positive fibers at the striatal level (Figure 1B). Hαsyn is observed along the axons from neurons of the SN, terminating in the STR. Expression of hαsyn is detected from the most anterior to the most posterior extent of the STR. This event seems to happen rapidly after the viral injection since expression of the transgenes in the terminals is observed after 1 week. (Figure 1B, injected side). Moreover, these data demonstrate that the luciferase reporter does not affect the ability of hαsyn protein to be transported along the nigrostriatal pathway.

**Figure 2:**
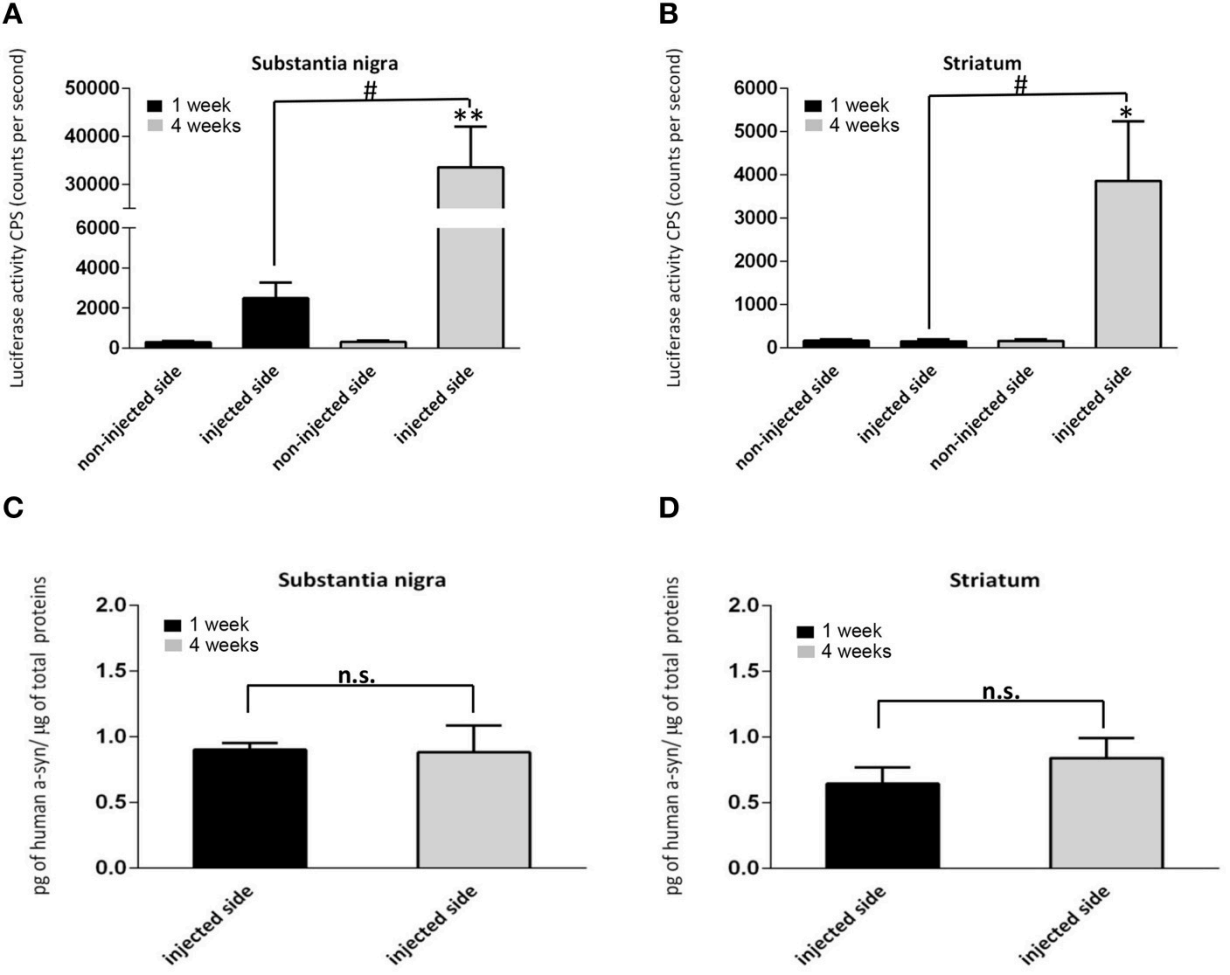
**Monitoring of αsyn oligomerization *ex vivo* using protein complementation assay. (A,B)** αsyn oligomers were quantified via luciferase assay in brain homogenates at 1 and 4 weeks after stereotaxic injection of SL1 and SL2. Signal detected in the non-injected side was used as a baseline. **(A)** In the SN, αsyn oligomers were detected at 1 week, increasing significantly by 4 weeks [*n* = 6 per group, Student *t*-test, *T*_(10)_ = 3.6; ^**^p < 0.001]. **(B)** In the STR no luciferase activity was detected at 1 week, but were observed by 4 weeks [*n* = 6 per group, Student's *T*-test, *T*_(10)_ = 2.4; ^*^p < 0.05]. **(C,D)** represent the total expression of h-αsyn assessed by ELISA in the homogenates of the SN **(C)** and STR **(D)** at 1 and 4 weeks. The data are expressed as the amount of h-αsyn normalized to the total amount of proteins in the homogenates. Statistical analysis showed no significant differences across the time for each regions (*p* > 0.05, *n* = 6 per group). In **(A,B)** number sign indicates significant differences between the injected side at 1 week and 4 week (^#^*p* < 0.05). n.s, not significant.

Because the substrate for hGluc, coelenterazine, does not permeate the blood brain barrier, *in vivo* luciferase signal was measured in homogenates *ex vivo* from the SN and STR overexpressing tagged hαsyn at 1 week (*n* = 7) and at 4 weeks (*n* = 8) post injection. In each group, all brains were processed at the same time and under the same conditions. During homogenization no detergent, no intense shaking, and no sonication are used. The tissue is briefly homogenized in PBS with a tissue grinder pestle and vortexed for 1 min. The contralateral non-injected sides (STR and SN) were processed in the same manner and served as internal controls for non-specific background signal. Interestingly, luciferase activity could be detected in the injected SN as early as 1 week post injection (Figure 2A), increasing dramatically by 13-fold at 4 weeks (Figure 2A). By contrast, in the STR, no luciferase activity is detected at 1 week post injection (Figure 2B) whereas, abundant luciferase activity is detected at 4 weeks (Figure 2B). The luciferase signal in tissue homogenate from injected animals indicates *in vivo* luciferase complementation, the surrogate marker of αsyn-αsyn interactions. As expected, in animals injected with only one half (AAV-SL2 only), no luciferase activity was detected after 4 weeks (Supplementary Figures 1A,B) demonstrating that fragmented luciferase does not give rise to aberrant luminescence signal. A second control incorporated into our experimental paradigm was a group of animals injected with one half of the complementation pair (AAV-SL1) in the left SN and the other half (AAV-SL2) in the right SN. After 4 weeks, homogenates from left and right SN or STR were mixed together and assayed for luciferase activity. As expected, no luciferase activity was detected (Supplementary Figure 1C) demonstrating that αsyn-αsyn interactions occur *in vivo* and not *ex-vivo* during tissue processing.

Reconstitution of luciferase activity *in vivo* demonstrates the successful complementation of luciferase halves via the formation of αsyn oligomers. Importantly, our data show the presence of αsyn oligomers in the SN as early as 1 week post-delivery of AAV-SL1 and AAV-SL2 (Figure 2A). The total level of hαsyn in the SN of the animals was quantified by ELISA at 1 and 4 weeks (Figure 2C). No significant difference was detected in hαsyn levels 1 and 4 weeks (Figure 2C, 1 week = 0.90 ± 0.05 4 weeks = 0.88 ± 0.22, *p* > 0.05). Interestingly, although equivalent levels of hαsyn was detected in SN at 1 and 4 weeks, the oligomeric profile was very different (Figure 2A). No hαsyn oligomers were detected in the STR at 1 week (Figure 2B) despite abundant expression of αsyn by ELISA quantification (Figure 2D). There are two ways these data can be interpreted; αsyn oligomers form in the SN and are then transported to the striatal terminals which takes longer than a week or that oligomers form at the terminals over a longer period of time (Figure 2B). Regardless, the data support the fact that sub-cellular location affects αsyn oligomerization kinetics.

### SL1 and SL2 overexpression induce progressive nigral and striatal dopaminergic neurodegeneration

**Figure 3:**
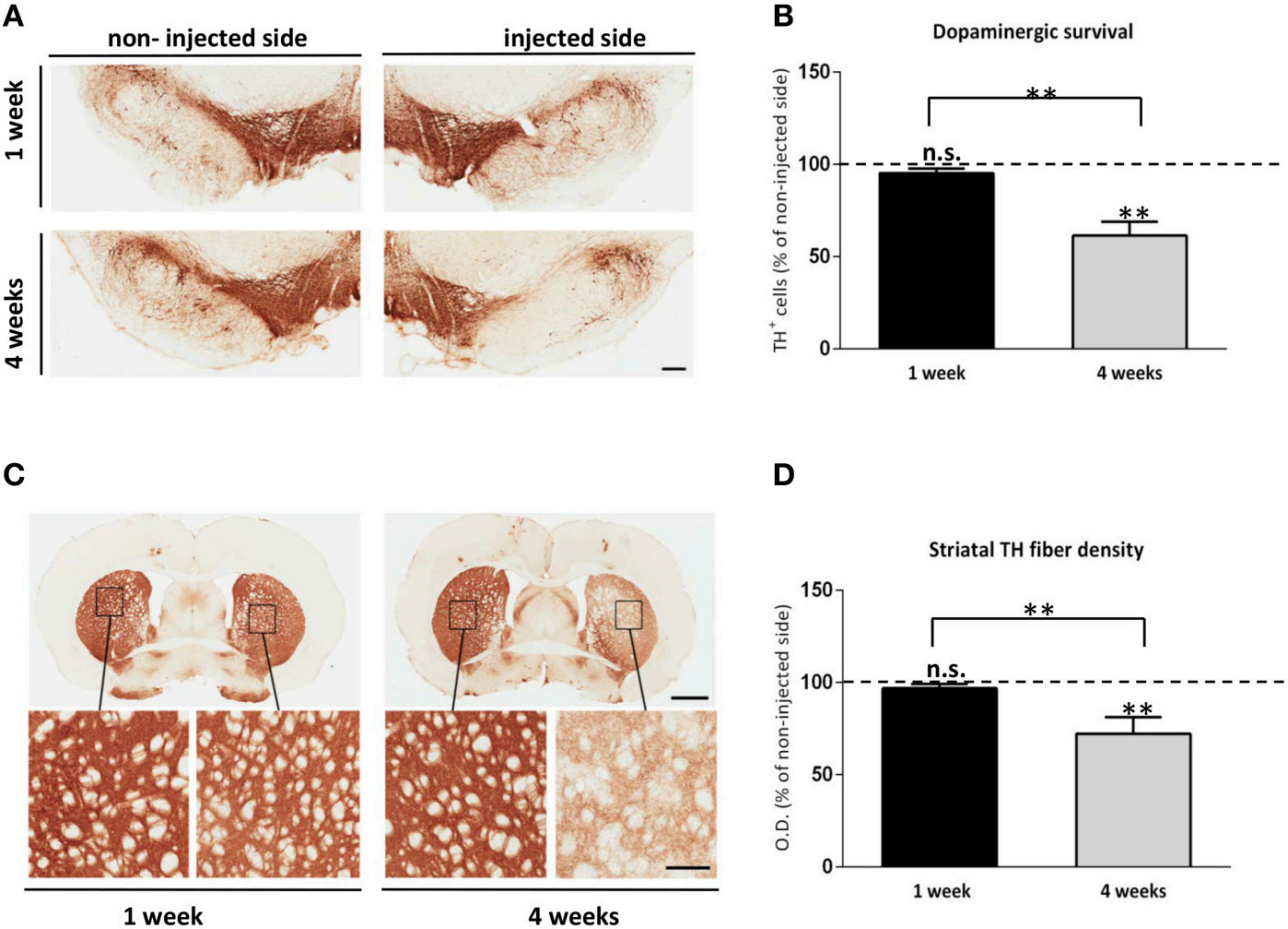
**Alpha synuclein associated neurotoxicity in the SL1SL2 rat model. (A)** Representative TH immunohistochemical staining of nigral sections in animals injected with AAV8-SL1SL2 at 1 and 4 weeks. Scale bar represents 250 μm. **(B)** illustrates in a graph bar the estimation of total TH+ cells in the SN (expressed as % of non-injected side) using unbiased stereological counting at 1 and 4 weeks. 38.5 ± 7.5% significant dopaminergic cell loss was found at 4 weeks when compared to the non-injected side [Paired Students' *T*-test, *T*_(9)_= 5.0, ^**^p < 0.001] and One-way ANOVA analysis followed by a Tuckey's for multiple testing revealed a 33.81 ± 7.9% significant difference between 1 and 4 weeks (^**^p < 0.001). **(C)** Photomicrographs illustrating TH+ terminals in the STR in SL1SL2 animals. Scale bars represent 2 mm and 300 μm (zoomed-in pictures). **(D)** Illustrates in a bar graph the quantitative densitometry of the TH+ fibers measurements (expressed as percentage of non-injected side). The bars represent the average of four different rostro caudal levels of the STR measured for each animal. Statistical difference was found at 4 weeks in the injected side compared to the non-injected side [Paired Students' *T*-test, *T*_(9)_ = 5.0, ^**^p < 0.001] and also between time point group (^**^p < 0.001). *n* = 6, for 1 week group and *n* = 8, for 4 weeks group.

Previous viral vector rodent models of αsyn overexpression have described a progressive nigral cell loss and striatal terminal neurodegeneration (Kirik et al., 2002; Klein et al., 2002). To confirm that our *in vivo* αsyn-αsyn bioluminescent complementation model exhibits similar characteristics, we immunostained coronal sections at the level of the SN and STR with an antibody against tyrosine hydroxylase (TH), and performed unbiased stereology and densitometry analysis. At 1 week, TH immunostaining and stereology of the SN indicated no loss of TH+ neurons in the injected side overexpressing SL1 and SL2 when compared to the non-injected side (Figure 3A). However, by 4 weeks a general reduction in the dendritic arborization in the SN of the animals was observed (Figure 3A) with stereological quantification confirming a 38.5 ± 7.5% dopaminergic cell loss in animals expressing SL1 and SL2 (Figure 3B). Also, comparison of nigral neurons at 1 and 4 weeks revealed a significant difference in the extent of cell loss between the groups. Dopaminergic cell death was further corroborated by the loss of fibers in the STR (Figures 3C,D). Densitometric analysis of TH stained sections showed a dense innervation throughout the STR of the non-injected side at all-time points. This was also true of the injected side at 1 week (Figures 3C,D OD black bar = 96.9 ± 2.2%). By contrast, at 4 weeks the injected side had a significant reduction of striatal TH+ fibers of 27.7 ± 7.4% relative to the contralateral side (Figure 3D). Thus, these data demonstrate a progressive cell and terminal loss through 4 weeks.

### Modulation of αsyn oligomerization in the rodent bioluminescent PCA model

The fact that αsyn oligomers can be detected in the SN as soon as 1 week post virus delivery make this a very attractive model for rapid screening of modulators of αsyn oligomers. Therefore, we performed a proof-of-concept experiment whereby rats were treated with a previously described heat shock protein inhibitor, SNX-9114 (McFarland et al., 2014). *In vitro* αsyn-induced aggregation and toxicity is prevented by inhibition of Hsp90 (Klucken et al., 2004; McLean et al., 2004; Danzer et al., 2011; Jones et al., 2014). Hsp90 inhibition results in activation of heat shock factor 1 and by consequence an up-regulation of protective stress-induced proteins such as Hsp70. We recently showed a novel class of Hsp90 inhibitors that could significantly reduce αsyn oligomerization in a cellular model of αsyn aggregation (Putcha et al., 2010; McFarland et al., 2014).

**Figure 4:**
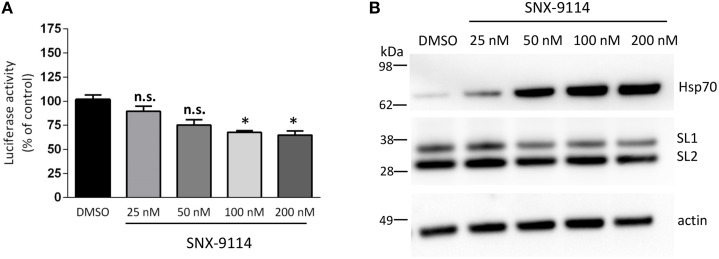
**Effect of SNX-9114 on αsyn oligomerization in cell culture. (A)** A range of concentrations of SNX-9114 was added to SL1SL2 cells stably overexpressing hαsyn for 24 h. The extent of intracellular αsyn oligomers was evaluated by luciferase assay and was normalized to DMSO control. Oligomerization was significantly decreased with a 100 and 200 nM doses (^*^p < 0.05). **(B)** Induction of Hsp70 after treatment with SNX-9114 on SL1SL2 cells was monitored by western blot as well as the expression of h-αsyn. Actin was used as a loading control. Data are given as mean ± S.E.M., from three independent experiments. Statistical analysis: One-way ANOVA followed by Tuckey's multiple comparison test. n.s., not significant.

Here we chose to use the Hsp90 inhibitor to validate our 1 week oligomers model. Because SNX-9114 has not been previously validated *in vitro*, we first evaluated its ability to reduce αsyn oligomer formation in our *in vitro* complementation assay. Stable cells expressing SL1 and SL2 were plated in a 96-well plate format and treated with 0.01% DMSO or increasing concentrations of the SNX-9114 (25, 50, 100, and 200 nM). Twenty-four hours later, cells were assayed for luciferase activity to determine the effect of the inhibitor on αsyn oligomerization as measured by reconstituted luciferase activity. A decrease in luciferase activity was first detected at 25 nM and 50 nM (Figure 4A, *p* > 0.05), however, at higher doses (100 and 200 nM) SNX-9114 significantly reduced αsyn oligomerization by 32.48 ± 2 and 35.1 ± 4.2% respectively compared to DMSO (Figure 4A, *p* < 0.05). Biochemical analysis of cells expressing SL1SL2 and treated with SNX-9114 confirmed the up-regulation of Hsp70 in a dose-dependent manner (Figure 4B) with little to no effect on steady state levels of αsyn except at the highest doses (Figure 4B).

**Figure 5:**
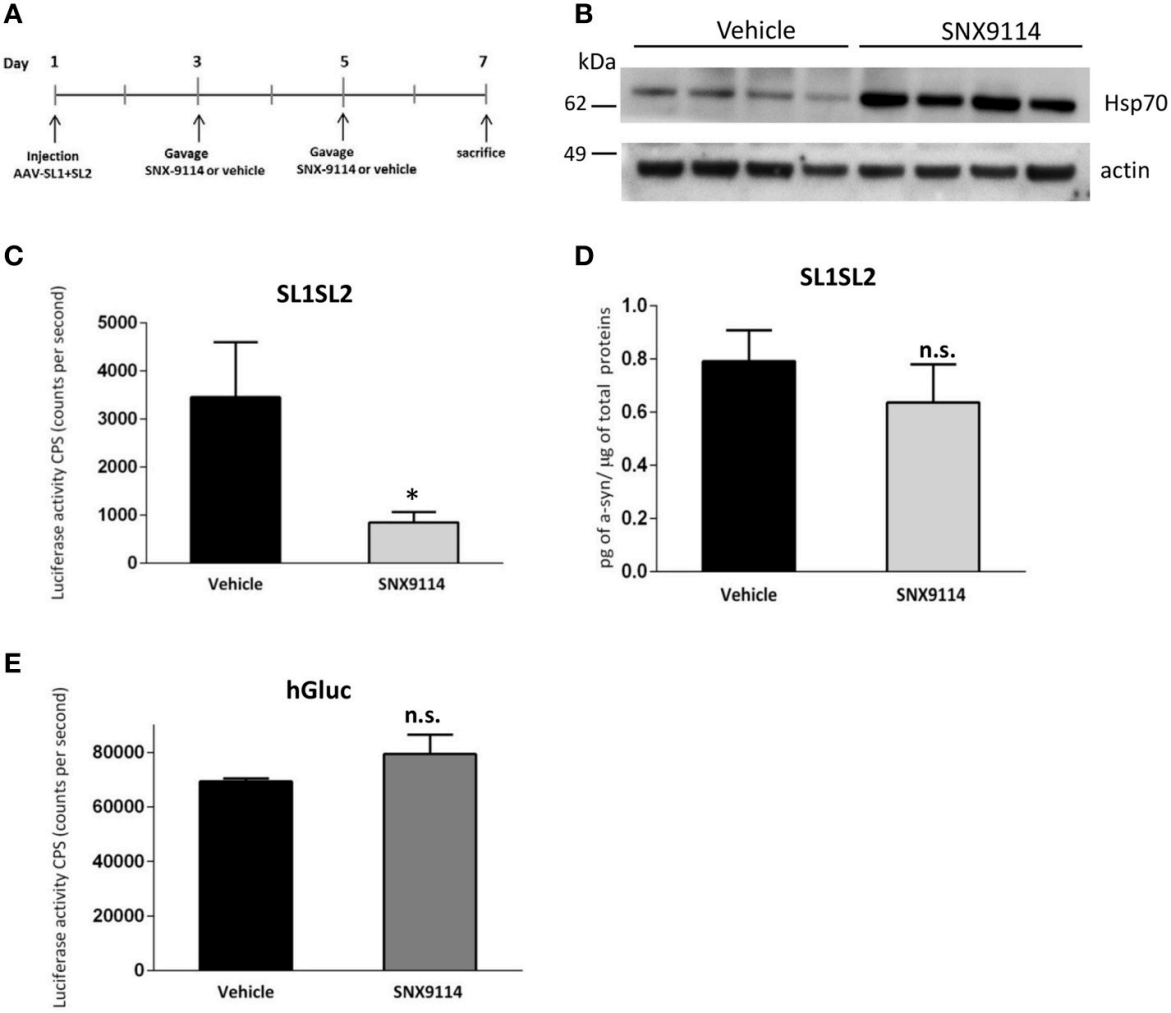
**Effect of SNX-9114 in SL1SL2 rat substantia nigra. (A)** Illustration of the timeline experiment of SNX-9114 treatment *in vivo*. **(B)** Hsp70 induction in the SN was evaluated by western blotting and actin was used as a loading control. Western blot shows four representative animals for each group. **(C)** Level of oligomers in the SN of the vehicle group (*n* = 6) and the treated group (*n* = 6) was assessed by luciferase activity 1 week after viral vector delivery. A significant decrease of αsyn oligomerization was observed after 2 mg/kg SNX-9114 treatment [Student's *T*-test, *T*_(9)_ = 2.46; ^*^p < 0.05]. **(D)** Expression of hαsyn in injected SN homogenate was analyzed by ELISA and is expressed as amount of hαsyn normalized to the total amount of protein in the homogenates. **(E)** Control rats injected with hGluc and treated either with vehicle (*n* = 2) or SNX-9114 (*n* = 2). The luciferase activity was not affected by the drug treatment as *p* > 0.05. n.s, not significant.

To determine if Hsp90 inhibition could reduce αsyn oligomer formation *in vivo* after 1 week, we stereotactically injected AAV-SL1 and AAV-SL2 into rat SN as described previously. Forty-eight hours after surgery we orally gavaged the rats with 2 mg/kg SNX-9114 or vehicle control. A second gavage of 2 mg/kg SNX-9114 or vehicle was performed 2 days later, and the rats were sacrificed at 7 days post-surgery (Figure 5A) A control group of rats transduced with AAV-hGluc were included to control for drug effects on the luciferase enzymatic reaction.

Western blot of homogenates prepared from the SN of injected rats confirmed the induction of Hsp70 in animals treated with SNX-9114 compared to animals treated with vehicle (Figure 5B). Furthermore, a luciferase activity assay on the same homogenates demonstrated a significant decrease in αsyn oligomers in the group of animals receiving the Hsp90 inhibitor (Figure 5C). No significant difference in hαsyn protein levels was detected between the drug and vehicle control groups when measured by ELISA (Figure 5D, *p* > 0.05). Of note, consistent with our *in vitro* experiments (Figure 4B), SNX-9114 did not directly affect the enzymatic luciferase signal *in vivo* (Figure 5E). These findings suggest that the decreased luciferase signal in the Hsp90 inhibitor treated group is indicative of less αsyn oligomer formation (Figure 5C).

## Discussion

Neuropathologic and genetic studies have provided strong evidence for the involvement of aggregated αsyn in the pathogenesis of synucleopathies (Tu et al., 1998; Dickson et al., 1999; Narhi et al., 1999; Masliah et al., 2000). Our present study proposes a rapid model for tracking αsyn oligomerization in selected brain regions of the rat brain using an *ex vivo* bioluminescent PCA.

Herein we demonstrate that, our split hGluc AAV-SL1 and SL2 vectors can be utilized to track αsyn oligomerization *in vivo*. As early as 1 week, we observe the expression of the transgenes along the nigrostriatal pathway and αsyn oligomers in the SN (Figures 1, 2). At this time point no toxicity is detected in the SN and no loss of dopaminergic fibers at the striatal level (Figure 3). The expression of hαsyn and presence of oligomers in SN but no cell loss as early as 1 week post-transduction make this an attractive model with which to screen for novel oligomerization modulators. Because no significant difference was detected in total αsyn levels between SN and STR at 1 week, the absence of αsyn oligomers in STR suggests distinct dynamics of oligomer formation in striatum compared to SN or alludes to oligomer formation occurring in the soma and subsequent trafficking of oligomers to the terminals where additional oligomer formation is seeded. Differentiating between these two mechanisms will require further investigation.

By 4 weeks, abundant αsyn oligomers are detected in both SN and STR and cell loss is detected in the SN, with terminal loss detected in the STR. αSyn expression in the SN and STR did not increase even though there was a 13-fold increase in luciferase activity. These data could support a hypothesis whereby increased asyn oligomers correlate to neurotoxicity, however further studies will be required before this can be confirmed.

Given the essential role of αsyn oligomers in the pathogenesis of synucleopathies it is essential that we develop reliable methods for their detection. Oligomer-specific αsyn antibodies (Emadi et al., 2009; Fagerqvist et al., 2013; Maetzler et al., 2014) recognizing relevant pathology have been designed and are relevant for immunohistochemistry of αsyn brain pathology on human or transgenic mouse (Delenclos et al., 2015; Sengupta et al., 2015). They also hold potential as therapies and could be a relevant disease biomarkers. However, such antibodies cannot monitor the oligomeric state of αsyn in a live cell environment. PCA strategies using fluorescence or bioluminescence reporters have been developed and have proven to be a powerful approach to study protein aggregation. Recently, we generated a rodent model using YFP venus PCA to monitor αsyn oligomerization in live animals (Dimant et al., 2014). Using fluorescence as readout, we directly detected αsyn oligomers and monitored αsyn aggregation in cortical neurons of living mice using two photon microscopy. Recently, a mouse model expressing firefly luciferase tagged-αsyn *in vivo* was generated and bioluminescent imaging (IVIS) was used to non-invasively capture αsyn oligomerization in the mouse brain (Aelvoet et al., 2014). Both of these *in vivo* models have distinct advantages such as the ability to follow the response in the same animals over time, providing a kinetic readout of the oligomerization process. In our model we measure the oligomerization *ex-vivo*. Although, all brains were processed at the same time and under the same conditions, we cannot exclude the possibility that the extraction procedure could perturb αsyn aggregation state. On the other hand, even though in *vivo* imaging represents a good tool to monitor αsyn, these methods require expensive instrumentation, are time-consuming, and involve anesthesia and systemic substrate injections in the case of the firefly luciferase PCA. Lastly, the two photon microscopy is limited by photo absorption of the tissue and does not allow deep brain structure imaging.

The model presented herein has the advantage of having a rapid readout without expensive equipment. HGluc may also provide increased sensitivity as it is over 100-fold more sensitive than the commonly used luciferases, Firefly, and *Renilla reniformis* (Tannous et al., 2005; Remy and Michnick, 2006). Also Gluc is a smaller reporter (185 amino acid; Tannous et al., 2005) compared to the other forms of luciferase or the fluorescence reporter. Finally, hGluc PCA is a fully reversible interaction unlike the assay based on YFP venus fluorophore, thus facilitating the detection of the kinetics of protein complex assembly/disassembly *in vivo*. Better spatial and temporal resolution can be achieved compared with *in vivo* imaging and we believe that hGluc PCA may be more efficient and effective for some approaches. In our current experiment, oligomers were quantified *ex vivo* for several reasons. Brain lysate analysis provides a more accurate semi quantitative measure of aggregates and most importantly, allows a combination of different analyses on the same sample (e.g., ELISA, WB) for each brain. Because the PCA assay is effective in tissue directly placed in a micro titer plate, its utility to measure oligomers in *in vivo* brain slice preparations is also a possibility. Also, it is well established that αsyn oligomers can be transmitted from neuron(s) to neuron(s), thus inducing pathology along neuroanatomical pathways in the brain (El-Agnaf et al., 2006; Emmanouilidou et al., 2010). It remains to be determined if αsyn induces neurodegeneration via intracellular- or extracellular-associated mechanisms. Our AAV-model, coupled with *in vivo* microdialysis, could be a useful tool for the detection and analysis of αsyn oligomers in the interstitial fluid (ISF) of living animals, allowing a real time monitoring of *in vivo* processes and opening possibilities for αsyn transmission studies. One disadvantage is that coelenterazine, the substrate of hGluc, does not cross the blood brain barrier and therefore excludes the use of our model for imaging in live animals. Despite this limitation, the use of hGluc PCA *in vivo* provides a rapid model to track αsyn oligomers depending on the desired readout.

To investigate the potential of our *in vivo* bioluminescent PCA to identify compounds that interfere with αsyn-αsyn interaction or modulate oligomerization, in a little as 1 week, we administered a cohort of AAV SL1 and SL2 transduced rats with 2 mg/kg SNX-9114, for one week. Hsp90 inhibition has been shown to modulate αsyn aggregation and toxicity (Auluck et al., 2002; Kalia et al., 2010; Luo et al., 2010; Putcha et al., 2010) and we have previously established that pharmacological pretreatment with geldanamycin, a naturally occurring Hsp90 inhibitor, protects against αsyn-induced toxicity and leads to degradation of high molecular-weight species (McLean et al., 2004) More recently we described the efficacy of novel brain permeable Hsp90 inhibitors to reduce the formation of αsyn oligomeric species in a dose dependent manner using our bioluminescent PCA system in H4 neuroglioma cells (Putcha et al., 2010). In line with these previous data, we show here that an Hsp90 inhibition can inhibit αsyn oligomerization in our AAV-SL1 and AAV-SL2 rodent model, and most importantly, we demonstrated that our luciferase assay is sensitive enough to detect these changes. In addition it is noteworthy to mention that the level of oligomers was assessed as early as 1 week after the AAV-injection, at a time when we do not detect cell death (Figure 3B) but we have an abundance of oligomers in the SN (Figure 2A). Therefore, the detected decrease in luciferase activity at 1 week in SN in animals receiving Hsp90 inhibitor, can be directly attributed to a decrease of αsyn oligomers.

While several strategies have been developed to monitor the modulation of αsyn aggregation *in vivo* and *in vitro*, the major advantage of the present approach over other techniques is the ability to quantify a change in αsyn oligomerization in a short period of time (1 week) with a fast, simple, and sensitive assay. In conclusion, our bioluminescent *in vivo* model represents a powerful new tool to study spatial and temporal changes in αsyn aggregation in response to new therapeutic agents that modulate αsyn oligomerization.

## Author contributions

MD carried out the study design, animal procedures, histology, analysis, and drafting of the manuscript. TT performed immunohistochemistry, stereology analysis, densitometry analysis, and participated in all animal procedures. DJ, participated in the study design, animal procedures, and editing of the manuscript. SM and AB performed the *in vitro* assay. MY, assisted with animal procedures. WH provided the compound SNX-9114. PM participated in the experimental design, coordination, interpretation, drafting, and editing of the manuscript. All authors read and approved the final manuscript.

### Conflict of interest statement

The authors declare that the research was conducted in the absence of any commercial or financial relationships that could be construed as a potential conflict of interest.

## Supplementary material

Supplementary Figure 1 **Luciferase activity in control rats. (A)** Animals were stereotactically injected with one viral vector (AAV8-SL2, *n* = 2). After 4 weeks, the luciferase activity was measured. No specific signal was detected in the injected side since the luciferase activity was similar to the non-injected side. In both regions, SN **(A)** and STR **(B,C)** To verify that the complementation occurs *in vivo* and not during homogenization process, animals were injected with AAV8-SL1 In the left SN and with AAV-SL2 in the right SN (*n* = 2). At 4 weeks homogenate of SN left and right or STR left and right, were mixed together respectively and luciferase activity was measured. No activity was detected in the mixture of SN or STR.
