# Supplementary figures and images for: A Rapid, Semi-Quantitative Assay to Screen for Modulators of Alpha-Synuclein Oligomerization Ex vivo
Source: Front Neurosci. 2016 Jan 19;9:511. doi: 10.3389/fnins.2015.00511 (PMC4717311; doi:10.3389/fnins.2015.00511)

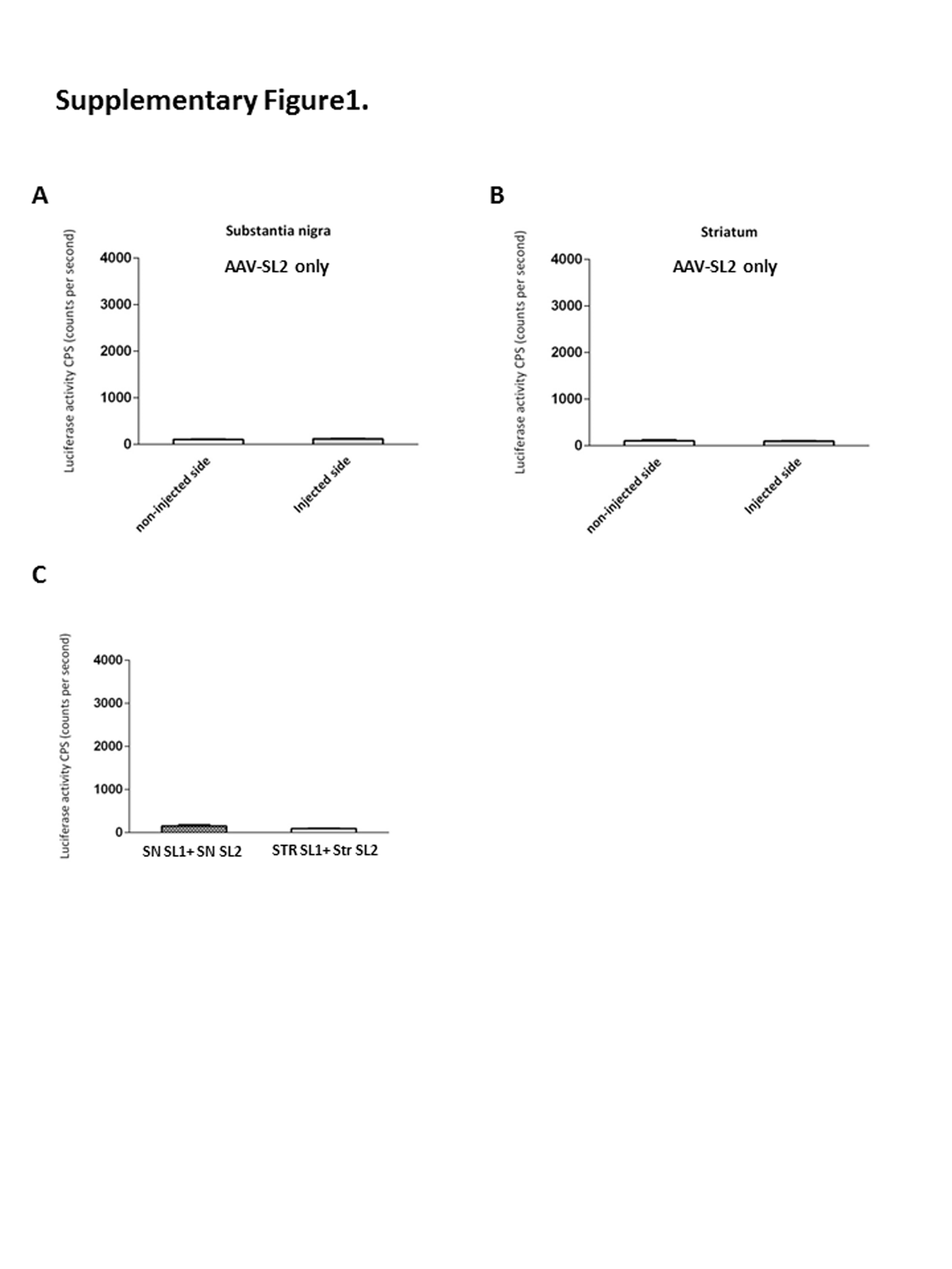

Supplement: Supplementary Figure 1 — Luciferase activity in control rats. (A) Animals were stereotactically injected with one viral vector (AAV8-SL2, n = 2). After 4 weeks, the luciferase activity was measured. No specific signal was detected in the injected side since the luciferase activity was similar to the non-injected side. In both regions, SN (A) and STR (B,C) To verify that the complementation occurs in vivo and not during homogenization process, animals were injected with AAV8-SL1 In the left SN and with AAV-SL2 in the right SN (n = 2). At 4 weeks homogenate of SN left and right or STR left and right, were mixed together respectively and luciferase activity was measured. No activity was detected in the mixture of SN or STR. [file Image1.tif]
